# Intimate partner violence, forced first sex and adverse pregnancy outcomes in a sample of Zimbabwean women accessing maternal and child health care

**DOI:** 10.1186/s12889-018-5464-z

**Published:** 2018-05-03

**Authors:** Simukai Shamu, Stephen Munjanja, Christina Zarowsky, Patience Shamu, Marleen Temmerman, Naeemah Abrahams

**Affiliations:** 1 Foundation for Professional Development, 173 Mary Road, The Willows, Pretoria, 0184 South Africa; 2 School of Public Health, University of the Witwatersrand, Johannesburg, South Africa; 3 Department of Obstetrics and Gynaecology, College of Health Sciences, University of Zimbabwe, Harare, Zimbabwe; 4 University of Montreal Hospital Research Centre, Montreal, QC, Canada; 5 School of Public Health, University of the Western Cape, Bellville, 7535 South Africa; 6 Wits Reproductive Health and HIV Institute, University of the Witwatersrand, Johannesburg, South Africa; 7 International Centre for Reproductive Health, Ghent University, 9000 Ghent, Belgium; 8 Gender and Health Research Unit, Medical Research Council, Cape Town, South Africa

**Keywords:** Intimate partner violence, Forced first sex, Maternal and newborn health outcomes, Zimbabwe

## Abstract

**Background:**

Intimate partner violence (IPV) remains a serious problem with a wide range of health consequences including poor maternal and newborn health outcomes. We assessed the relationship between IPV, forced first sex (FFS) and maternal and newborn health outcomes.

**Methods:**

A cross sectional study was conducted with 2042 women aged 15–49 years attending postnatal care at six clinics in Harare, Zimbabwe, 2011. Women were interviewed on IPV while maternal and newborn health data were abstracted from clinic records. We conducted logistic regression models to assess the relationship between forced first sex (FFS), IPV (lifetime, in the last 12 months and during pregnancy) and maternal and newborn health outcomes.

**Results:**

Of the recent pregnancies 27.6% were not planned, 50.9% booked (registered for antenatal care) late and 5.6% never booked. A history of miscarriage was reported by 11.5%, and newborn death by 9.4% of the 2042 women while 8.6% of recent livebirths were low birth weight (LBW) babies. High prevalence of emotional (63,9%, 40.3%, 43.8%), physical (37.3%, 21.3%, 15.8%) and sexual (51.7%, 35.6%, 38.8%) IPV ever, 12 months before and during pregnancy were reported respectively. 15.7% reported forced first sex (FFS). Each form of lifetime IPV (emotional, physical, sexual, physical/sexual) was associated with a history of miscarrying (aOR ranges: 1.26–1.38), newborn death (aOR ranges: 1.13–2.05), and any negative maternal and newborn health outcome in their lifetime (aOR ranges: 1.32–1.55). FFS was associated with a history of a negative outcome (newborn death, miscarriage, stillbirth) (aOR1.45 95%CI: 1.06–1.98). IPV in the last 12 months before pregnancy was associated with unplanned pregnancy (aOR ranges 1.31–2.02) and booking late for antenatal care. Sexual IPV (aOR 2.09 CI1.31–3.34) and sexual/physical IPV (aOR2.13, 95%CI: 1.32–3.42) were associated with never booking for antenatal care. Only emotional IPV during pregnancy was associated with low birth weight (aOR1.78 95%CI1.26–2.52) in the recent pregnancy and any recent pregnancy negative outcomes including LBW, premature baby, emergency caesarean section (aOR1.38,95%CI:1.03–1.83).

**Conclusions:**

Forced first sex (FFS) and intimate partner violence (IPV) are associated with adverse maternal and newborn health outcomes. Strengthening primary and secondary violence prevention is required to improve pregnancy-related outcomes.

## Background

Violence against women is a serious violation of the human and women’s rights with substantial effects on maternal and child morbidity and mortality [1, 2]. Intimate partner violence during pregnancy impacts on both mother and child health. Negative health outcomes include unintended pregnancies, STI/HIV infection, maternal injuries or death and negative effects to the baby including preterm birth, abortion, low birth weight, neonatal death, HIV infection. Poor access to maternity care due to the effects of violence could influence all of the above health outcomes, [3–7]. Until recently, the most relevant evidence came from western/high-income countries, the WHO Multi country study on violence [8, 9], and the Demographic and Health Survey (DHS) series [10, 11]. The evidence from developing countries is emerging. Most previous studies on IPV and maternal health effects did not assess the relationship between individual types of IPV and maternal and newborn health effects. More studies are needed to assess the magnitude of the relationship between IPV and pregnancy outcomes in low income countries. In this paper we present an analysis which assesses both individual and combined types of violence to establish the different associations between IPV types and individual and combined maternal and newborn health effects.

Zimbabwe is one of the countries with the highest rates of maternal health challenges. While the global average maternal mortality rate was 210 per 100,000 live births, maternal mortality in Zimbabwe was 570/100000 live births in 2010, above the average sub-Saharan African rate of 500 [12]. Reasons for negative maternal and newborn health outcomes have been explored broadly under the “three delays” model: delay to decide to seek care, to reach care and to receive treatment [13]. This conceptualisation of delays is limited in explaining negative health outcomes as it does not specifically target violence and gender inequalities as causes of negative maternal and neonatal health effects.

Between 5 and 46% women report forced first sexual intercourse (FFS) in Sub-Saharan Africa [14]. Some studies have found FFS to be associated with sexually transmitted infections (STIs) and unintended pregnancies among young people [15] while others reported associations with HIV/STIs, multiple and high-risk sex partners and non-condom use [14]. Most studies assessing effects of forced first sex were conducted among adolescents aged 14–24 years [16–18], focussing on HIV infection, and pregnancy. Among the studies with pregnant women [19] only a few assessed outcomes beyond HIV during the current pregnancy while previous outcomes are not included.

In this paper we present an analysis of the relationship between IPV and maternal and newborn health outcomes. We explored forced first sex, lifetime IPV, IPV in the last 12 months before the recent pregnancy and IPV during pregnancy and looked for each type or combined types’ associations with adverse maternal and newborn effects. We hypothesised that women who experienced forced first sex or IPV will be at higher risk of experiencing adverse maternal and newborn health outcomes than women experiencing no violence. Directly, if one is physically abused during pregnancy, the violence may injure the mother and/or the foetus or forced sex may result in unplanned pregnancy. Indirectly, abuse such as emotional abuse may lead to depression [20] which is associated with low birth weight [21, 22] and other negative consequences. An indirect effect of forced first sex may be that the abused woman or girl would be socialised to accept the use of violence to enforce discipline and as such tolerate violence in later life leading to poor health including maternal and child health [23]. The analysis will test the relationship between violence (forced first sex and IPV) and adverse maternal and child health effects.

## Methods

A cross sectional study was conducted with 2042 women aged 15–49 years attending postnatal care at six public health clinics in low income neighbourhoods in Harare, Zimbabwe in 2011. The methodology for the study has been reported in detail elsewhere [24]. Data for the study were collected through face to face interviews and extracted from clinic records. The study sample was not population based but a clinic based sample in the main city. The sample was drawn from women attending postnatal clinics who were recruited while they were queuing for services. Face to face interviews were conducted in private. The clinics from which women were recruited had both antenatal and postnatal services and were owned and managed by the Harare City Council.

We measured lifetime and past year emotional, physical and sexual IPV before the recent pregnancy and during pregnancy using an adapted version of the questionnaire developed for the WHO Multi Country Study on Women’s Health and Domestic Violence against Women [25]. The questionnaire was validated in ten countries including in Africa. To determine lifetime IPV we asked behaviour specific questions such as “Has your current husband/partner, or any other partner ever hit you with his fist or with something else that could hurt you?” The response categories were dichotomised into Yes or No answers. For those who responded affirmatively, the following question was asked to measure IPV in the last 12 months before pregnancy: “Did this happen during the 12 months before the most recent pregnancy?” and lastly, “Did this happen during your most recent pregnancy?” to measure IPV during recent pregnancy. Six questions assessed physical IPV, while three questions assessed sexual and five questions assessed emotional IPV. Any one affirmative answer indicated that a person has experienced IPV. Forced first sexual intercourse was defined as reporting first sexual intercourse as forced against one’s will or being raped.

Maternal health outcomes were derived from the interviews as well as from clinic records. Some of the outcomes (e.g. HIV status) were verified across the two data sources. The maternal and newborn health outcomes included outcomes related to the current and the previous pregnancies. Outcomes related to previous pregnancies include: history of a miscarriage (miscarriage was defined as death of a foetus before 28 weeks of gestation); history of a stillbirth (giving birth to a baby that is dead after 28 weeks of pregnancy) and history of a neonatal death (giving birth to a live baby who died in the first 4 weeks of life). We also assessed gravidity (as total number of pregnancies in a lifetime) and age at first pregnancy. Maternal and newborn health outcomes on the most recent pregnancy included pregnancy planning, time of pregnancy booking for antenatal care and birth weight. To determine pregnancy planning women were asked if they planned their most recent pregnancy or not. We assessed gestation at time of booking for antenatal care. To determine if booking was delayed we categorised early booking as booking in the first or second trimester while late booking was considered as booking in the last trimester. A low birth weight was defined as a baby weighing less than 2500 g at birth. Women’s HIV status, history of smoking and alcohol drinking were assessed and considered as effect modifiers.

### Analysis

Data were analysed in Stata version 13 [26]. Frequencies were calculated. Maternal and neonatal health outcomes were described by experiences of IPV using chi-square tests to define differences between groups. Statistical differences were reported at 95% confidence levels. Univariate analyses were performed and different combinations of outcomes were performed before the multiple regression models were built. Three multiple logistic regression models were run to assess the relationships between FFS, IPV experiences at the level of lifetime, last 12 months before pregnancy and during pregnancy and maternal and newborn health outcomes. Regression model outputs were reported as adjusted Odds Ratios (aOR) and *p* values. We controlled for known covariates including demographic variables (age, marital status, education), HIV status and clustering of the sample as well as other known covariates depending on the model. For example, we controlled for smoking when assessing the relationship between low birth weight babies and violence during pregnancy since smoking is known to be a risk factor for low birth weight [27]. In assessing the relationship between IPV during pregnancy and pregnancy variables we also controlled for past year IPV to limit the effect of previous IPV experiences on current/recent outcome measures.

## Results

A total of 2101 women were approached to participate in the study of whom 2042 (97%) agreed and completed interviews. The mean age of the women interviewed was 26.4 years (standard deviation 5.71). Table 1 shows the prevalence of pregnancy and maternal outcome characteristics of the 2042 women interviewed. The mean age at first pregnancy was 20.2 years (standard deviation 3.03) years and the average gravidity was 2.2. Regarding previous pregnancies over one tenth and slightly under one tenth reported a history of miscarriage and newborn death respectively. Outcomes for the most recent pregnancy show that 27.6% pregnancies were not planned while over half (50.9%) of women booked late for antenatal care and more than one in 20 (5.6%) did not attend antenatal care at all. Mean birthweight was 3036 g while 8.6% women gave birth to low birth weight babies (< 2500 g).

**Table 1:** Maternal and child health outcomes of the sample (*N* = 2042)

	n	%	Mean	LCI	UCI
Maternal outcomes from most recent pregnancy					
Unplanned pregnancy (*n* = 2039)	657	32.2			
Early booking (*n* = 2038)	886	43.5			
Late booking (*n* = 2038)	1037	50.9			
Unbooked (*n* = 2042)	114	5.6			
Low birth weight (*n* = 2007)	173	8.6			
Mean birth weight in grammes (*n* = 2007)			3036	3016.66	3055.85
Premature (*n* = 2009)	31	1.54			
Caesarian Section (*n* = 2002)	65	3.25			
Extended hospital Stay (4+ days during delivery)	149	7.6			
Maternal outcomes from previous pregnancies					
Age at first pregnancy (mean) *n* = 2042			20.22	20.09	20.35
Gravidity (mean no. of lifetime pregnancies) (*n* = 2042)			2.22	2.17	2.27
History of miscarriage (*n* = 2039)	234	11.5			
History of a stilbirth (*n* = 2038)	30	1.37			
History of a premature baby (*n* = 2037)	35	1.72			
History of a neonatal death (*n* = 2007)	188	9.37			

The prevalence of intimate partner violence for this population was high across all types and is shown in Tables 2, 3 and 4. Table 2 shows the prevalence of lifetime emotional (64%), physical (37%) and sexual (52%) IPV and FFS (15.7%) by history of a neonatal death and miscarriages. More neonatal deaths and miscarriages were reported by women who reported being ever abused by their partners than by women who reported no abuse (*p* < 0.001). While strong associations were observed between experiences of all types of lifetime IPV, FFS and a history of neonatal death, miscarriage and/or stillbirth, no association between lifetime experiences of violence and maternal and newborn health outcomes in the recent pregnancy was observed.

**Table 2:** Prevalence of lifetime intimate partner violence by maternal health outcomes *N* = 2042

				Neonatal death					Miscarried (*N* = 2039)						
		Total abused		No (1818)		Yes (188)		Sig (*p*-value)	Total abused		No (1747)		Yes (292)		Sig (*p*-value)
		N	%	n	%	n	%		N	%	n	%	n	%	
Emotional IPV		1290	64.26	1162	63.88	128	68.09	0.25	1308	64.15	1145	63.43	163	69.66	0.062
Physical IPV		749	37.32	659	36.23	90	47.87	0.00	761	37.32	654	36.23	107	45.73	0.005
Sexual IPV		1038	51.72	919	50.52	119	63.30	0.00	1052	51.59	922	51.08	130	55.56	0.197
Sexual/Physical IPV		1306	65.07	1161	63.83	145	77.13	0.0001	1325	64.98	1165	64.54	160	68.38	0.247
Forced First Sex		314	15.69	278	15.33	36	19.15	0.171	314	15.45	272	15.12	42	17.95	0.26
Any history of negative outcome (ever had a neonatal death, miscarriage, and or stilbirth)									Any recent negative outcome Low birth weight, premature baby, c-section)						
		Total abused		No (1818)		Yes (188)		Sig (*p*-value)	Total abused		No (1818)		Yes (188)		Sig (*p*-value)
		N	%	n	%	n	%		N	%	n	%	n	%	
Emotional IPV		1308	64.15	1029	62.59	279	70.63	0.003	1291	64.17	1131	64.22	160	63.75	0.882
Physical IPV		761	37.32	578	35.2	183	46.33	0.0001	749	37.23	659	37.42	90	35.86	0.631
Sexual IPV		1052	51.59	824	50.12	228	57.72	0.007	1039	51.64	914	51.9	125	49.8	0.533
Sexual/Physical IPV		1325	64.98	1046	63.63	279	70.63	0.009	1306	64.91	1145	65.02	161	64.14	0.785
Forced First Sex		314	15.45	239	14.59	75	18.99	0.03	313	15.6	271	15.43	42	16.8	0.577

**Table 3:** Prevalence of IPV in the past 12 months before recent pregnancy by maternal and new born health outcomes

	Unplanned pregnancy (*N* = 2039)								
	Total abused		No (*n* = 1382)		Yes (*n* = 657)				
	N	%	n	%	n	%	Sig (*p*-value)		
Emotional IPV	822	40.3	516	37.3	306	46.6	0.000		
Physical IPV	434	21.3	260	18.8	174	26.5	0.000		
Sexual IPV	726	35.6	468	33.9	258	39.3	0.017		
Sexual/Physical IPV	944	46.3	608	44.0	336	51.1	0.002		
Forced First Sex	314	15.47	183	12.43	131	23.48	0.000		
	Any recent negative outcome (LBW, premature baby, c-section)								
	Total abused		No (1818)		Yes (188)		Sig (*p*-value)		
	N	%	n	%	n	%			
Emotional IPV	810	40.26	710	40.32	100	39.84	0.885		
Physical IPV	428	21.27	375	21.29	53	21.12	0.948		
Sexual IPV	718	35.69	634	36	84	33.47	0.433		
Sexual/Physical IPV	932	46.32	821	46.62	111	44.22	0.476		
Forced First Sex	313	15.6	271	15.43	42	16.8	0.577		
	Pregnancy Booking at antenatal care clinic								
	Total abused		Unbooked (*n* = 114)		Late booking (*n* = 284)		Early booking (*n* = 886)		
	N	%	N	%	n	%	n	%	Sig (*p*-value)
Emotional IPV	821	40.3	54	47.4	427	41.2	340	38.4	0.13
Physical IPV	434	21.3	24	21.1	258	24.9	152	17.2	0.00
Sexual IPV	725	35.6	59	51.8	368	35.5	298	33.6	0.001
Sexual/Physical IPV	943	46.3	70	61.4	499	48.1	374	42.2	0.0001
Forced First Sex	315	15.5	27	23.68	175	16.92	113	12.8	0.002

**Table 4:** Prevalence of IPV during recent pregnancy by maternal and new born health outcomes

			Low birth weight (*N* = 2007)				
	Total abused		No (*n* = 1834)		Yes (173)		
	N	%	n	%	n	%	Sig (*p*-value)
Emotional IPV	880	43.85	785	42.80	95	54.91	0.002
Physical IPV	319	15.89	284	15.49	35	20.23	0.10
Sexual IPV	782	38.96	709	38.66	73	42.20	0.36
Sexual/Physical IPV	928	46.24	842	45.91	86	49.71	0.34
Forced First Sex	305	15.24	273	14.93	32	18.6	0.199
Any recent negative outcome LBW, premature baby, c-section)							
	Total abused	No (1818)		Yes (188)			Sig (*p*-value)
	N	%	n	%	n	%	
Emotional IPV	881	43.79	756	42.93	125	49.8	0.04
Physical IPV	317	15.76	274	15.56	43	17.13	0.522
Sexual IPV	781	38.82	689	39.13	92	36.65	0.452
Sexual/Physical IPV	926	46.02	817	46.39	109	43.43	0.377
Forced First Sex	313	15.6	271	15.43	42	16.8	0.577
	Hospitalisation (*N* = 1974)						
	Total abused		No (*n* = 1825)		Yes (*n* = 149)		
	N	%	n	%	n	%	Sig (*p*-value)
Emotional IPV	866	43.87	798	43.56	71	47.65	0.333
Physical IPV	307	15.55	287	15.73	20	13.42	0.456
Sexual IPV	772	39.11	721	39.51	51	34.23	0.204
Sexual/Physical IPV	912	46.2	852	46.68	60	40.27	0.131
Forced First Sex	294	14.94	273	15.01	21	14.09	0.763

Table 3 shows the prevalence of last 12 months emotional (40%), physical (21%) and sexual (36%) IPV before pregnancy by maternal and newborn health outcomes. Women who reported negative maternal health outcomes (unplanned pregnancy and those who registered late for antenatal care or never registered at all) reported more violence (except for emotional violence) than women whose pregnancies were planned and who registered for care. Women who reported any negative outcomes in the most recent pregnancy (low birth weight, premature baby or emergency caesarean section) did not report more violence than those who reported no negative outcomes.

Table 4 shows the prevalence of emotional (44%), physical (16%) and sexual (39%) IPV during the most recent pregnancy by LBW and any negative outcomes in recent pregnancies, extended child birth related hospitalisation. More women who reported being emotionally abused during their pregnancy reported giving birth to low birth weight babies (*p* < 0.002) and any negative outcome (low birth weight, premature baby, emergency caesarean section) (*p* = 0.04) than women who did not report emotional abuse. There was no difference between abused and non-abused women by extended hospital stay in the recent pregnancy.

Table 5 shows results from the multiple regression analysis showing the association between different types of IPV and maternal and neonatal health outcomes. Women who experienced lifetime abuse had higher odds of reporting ever having a miscarriage or stillbirth in their lifetime. Forced first sex was not associated with ever miscarrying or having a stillbirth. Women who reported being abused in their lifetime had higher odds of reporting a neonatal death; reporting sexually/physically abused had the strongest relationship with neonatal death (aOR 2.05; 95%CI: 1.40–2.98).

**Table 5:** Multiple logistic regression analysis showing the association between IPV (ever, last 12 months before pregnancy and during pregnancy) and maternal and new born health outcomes

Association between types of lifetime IPV and maternal and new born health outcomes*												
	History of a Miscarry/stillbirth			History of a Neonatal death			History of a miscarriage			Ever had a neonatal death, miscarriage, and or stilbirth		
	aOR	95% CI	Sig (pvalue)	aOR	95% CI	Sig (pvalue)	aOR	95% CI	Sig (pvalue)	aOR	95% CI	Sig (*p*-value)
Emotional IPV	1.39	1.08–1.77	0.008	1.34	1.03–1.72	0.024	1.29	0.95–1.74	0.098	1.32	1.02–1.69	0.03
Physical IPV	1.38	1.06–1.79	0.0001	1.17	1.16–2.20	0.015	1.49	1.12–1.98	0.006	1.56	1.22–1.97	0.0001
Sexual IPV	1.34	1.03–1.74	0.029	1.80	1.29–2.49	0.000	1.17	0.88–1.55	0.274	1.40	1.10–1.76	0.005
Sexual/Physical IPV	1.26	0.95–1.67	0.0513	2.05	1.40–2.98	0.000	1.18	0.87–1.58	0.287	1.43	1.11–1.84	0.005
Forced First Sex	1.23	0.85–1.77	0.266	1.22	0.80–1.84	0.351	1.47	1.03–2.09	0.032	1.45	1.06–1.98	0.018
Association between IPV in the last 12 months and entry into care and maternal and new born health outcomes**												
	Unplanned pregnancy			Late booking (ref. = early booking)			Never booked (ref = early booking)			Any negative outcome (LBW, premature, still baby) in recent pregnancy		
	aOR	95% CI	Sig (pvalue)	aOR	95% CI	Sig (*p-*value)	aOR	95% CI	Sig (pvalue)	aOR	95% CI	Sig (*p*-value)
Emotional IPV	1.48	1.22–1.79	0.0001	1.11	0.91–1.33	0.287	1.43	0.89–2.28	0.287	1.03	0.77–1.38	0.8
Physical IPV	1.50	1.19–1.87	0.0001	1.57	1.24.1.97	0.0001	1.18	0.66–2.09	0.565	0.99	0.70–1.39	0.962
Sexual IPV	1.32	1.08–1.60	0.006	1.10	0.90–1.32	0.358	2.09	1.31–3.34	0.002	0.95	0.7–1.26	0.709
Sexual/Physical IPV	1.32	1.09–1.60	0.004	1.27	1.05–1.52	0.012	2.13	1.32–3.42	0.002	0.95	0.71–1.25	0.711
Forced First Sex	2.02	1.54–2.63	0.0001	1.36	1.03–1.78	0.028	1.37	0.74–2.51	0.312	1.04	.70–1.54	0.842
Association between IPV during pregnancy and recent new born health outcomes***												
	Recent Low birth weight			Any recent negative outcome (LBW, premature baby, c-section)								
	aOR	95% CI	Sig (pvalue)	aOR	95% CI	Sig (pvalue)						
Emotional IPV	1.79	1.26–2.52	0.001	1.38	1.03–1.83	0.026						
Physical IPV	1.23	0.78–1.91	0.362	1.09	0.74–1.59	0.673						
Sexual IPV	1.23	0.87–1.72	0.239	0.88	0.65–1.16	0.366						
Sexual/Physical IPV	1.22	0.87–1.70	0.246	0.89	0.67–1.17	0.406						
Forced First Sex	1.19	0.73–1.82	0.518									

*Model adjusted for age, education, marital status and HIV status

**Model adjusted for age, education, marital status, HIV status and history of smoking or alcohol use

***Model adjusted for age, education, marital status, HIV status and history of smoking, violence or alcohol use

We also assessed the relationship between being abused in the last 12 months before pregnancy and pregnancy planning, pregnancy booking and outcomes of pregnancy. Women who reported experiencing violence 12 months before the pregnancy had higher odds (aOR 1.32 - aOR 1.50) of reporting unplanned pregnancy compared to women who did not experience IPV. FFS was strongly associated with unplanned pregnancy (aOR 2.02). Results also show that booking late for pregnancy care was associated with being physically aOR 1.56; 95% CI 1.24–1.97) and sexually/physically (OR1.21) abused in the past 12 months before pregnancy but never booking at all was strongly associated with being physically (aOR 2.09 95% CI:1.31–3.34) and sexually/physically (aOR 2.13 95%CI 1.32–3.42) abused. Forced first sex was strongly associated with late booking.

We also assessed the relationship between IPV during pregnancy and recent pregnancy outcomes. Experiencing emotional (but not physical or sexual) IPV during pregnancy (aOR 1.76) was associated with a LBW baby. Emotional IPV was also associated with any negative outcome from the recent pregnancy.

## Discussion

This study confirms the association between intimate partner violence (sexual, emotional, sexual) and FFS and negative maternal and newborn health effects. To the best of our knowledge this is the first study on the relationship between IPV and maternal health outcomes in Zimbabwe. Previous studies have found associations between IPV and maternal health factors such as low birth weight, unintended pregnancy, preterm birth, still birth, abortion, miscarriage [28–35]. However, most of the studies were conducted outside the African continent with some studies having sample sizes lower than 500 participants. Our study included a wide range of pregnancy and newborn outcomes that span previous pregnancies and the most recent pregnancy. A further strength of the study was the inclusion of a wide range of violent experiences and indeed we found that women experienced high rates of FFS, IPV in their lifetime, last 12 months before pregnancy and during pregnancy.

Our study adds to the literature on the association between lifetime IPV and lifetime maternal health effects such as miscarriage or stillbirth. It goes further to highlight the relationship between different forms of IPV and FFS and both maternal and newborn outcomes. It also makes a unique contribution by assessing the types of pregnancy outcomes on the part of the mother as well as on the part of the newborn, firstly as single forms and later as combined forms of outcomes in relation to the single and combined forms of IPV and FFS. Such an assessment in one study helps to give a full picture of the relationship of IPV, FFS and pregnancy outcomes across one’s lifetime. The study illustrates the types of violence that affect certain health outcomes – individually and when combined.

Maternal and newborn health outcomes were poor in this population but consistent with previous trends. The mean birth weight (3036 g) was consistent with national estimates (3027 g) as reported in a population based study conducted in Zimbabwe [36]. While we found 8.6% LBW babies the ZDHS reported 14% children with low birth weight for Harare City, the city in which our study was conducted [37]. This may be due to our sample having a large proportion of women consulting skilled health workers in antenatal care compared to population based studies which include larger proportions of women not attending antenatal care clinics. Although the ZDHS was a population based study with the ability to capture a representative sample, the birth weight measure was subjective as it relied on a mother’s memory of weight for birth that took place up to the last 5 years before the interview which may have introduced more recall bias than our measure that relied on the birth weight as recorded in the mother’s card and clinic records for the recent births.

Generally, high levels of IPV during pregnancy were reported in our study. Although numerous arguments explaining why IPV is high and commonplace have been put forward [38], those that seem to explain the situation in Zimbabwe include that high rates of emotional violence may be a reflection of the babies being unplanned which triggered conflict leading to men to stress their partners. In addition, high sexual violence prevalence may be a reflection of the male partners’ failure to understand the pregnancy related changes that interfered with their need for continued sexual intercourse acts without matching the pregnancy situation limitations [39]. In addition we reported earlier in a qualitative study how service providers encourage women to endure sexual violence [40]. Such male power dominance is also seen in women reporting higher levels of partner control in Zimbabwe [39].

Only emotional violence was associated with low birth weight. Similarly, in one study physical abuse was not associated with low birth weight but only after regressing those who were injured through physical abuse against those who were not injured [41]. This may mean that physical violence is only associated with low birth weight if it is severe. Our analysis did not look at the severity of IPV in relation to low birth weight. However, since emotional violence does not usually occur in isolation, physical and sexual abuse may also have contributed towards low birth weight. Women who experience emotional abuse have more mental health problems such as depression which affect self-care. The interconnectedness of violence types must not therefore be ignored. Further studies can construct variables that include whether a woman was both abused physically and emotionally to assess the combined effect of such abuse. The causal pathways between emotional abuse and low birth weight need to be explained as this is not a direct cause and effect relationship. Emotional violence may cause women to become stressed and depressed which may negatively interfere with the regulation of the immune system and growth of the baby resulting in giving birth to low birth weight babies [42–44]. Stress and depression can also exacerbate chronic health problems like diabetes and hypertension for which many women are at risk [44]. We did not have data to assess this. Although depression may lead to unhealthy behaviours such as alcohol and drug abuse [45] which are known causes of low birth weight, our sample did not have many women who smoked prior to/or during pregnancy [24]. It may be necessary to screen for emotional IPV together with antenatal depression so that women with positive screens of IPV may be assisted in preventing chances of giving birth to low birth weight babies.

We reported a high rate of unintended pregnancy (32.2%). Similar rates of unplanned pregnancies (35.1% [46]; 32.1% [47] were reported in population based studies in Zimbabwe. Having almost a third of the women with an unmet need remains a cause for concern in a country that has been a global icon of family planning programming and implementation [48]. Given the high literacy and education rates in the study population and the high marketing of family planning methods in Zimbabwe, attributing unplanned pregnancies to relational and gender imbalance factors is not out of place since contraceptives are also readily available and provided free of charge. It is therefore possible that relationship factors that are evident through male power domination as evidenced by high rates of IPV in the sample explain high rates of unwanted pregnancies. Abused women have less negotiating power to protect themselves against falling pregnant. In such populations, strengthening efforts to educate men on equitable gender relations should be prioritised for the benefit of accessing and actually freely using contraceptives to prevent unwanted pregnancies. The relationship between IPV and unintended pregnancy has been explored, albeit using a lifetime IPV measure from the demographic and health surveys in several non-western countries including Colombia [4], India [49] and Bangladesh [50] and also western countries [34]. In order to avoid overestimating the relationship with IPV from multiple partners including partners who are not related to the current pregnancy Pallitto and colleagues only regressed the IPV by partners who were related to the pregnancy in question and found an association between IPV and unintended pregnancies [9]. Our study looked at the association between IPV that happened in the last 12 months before the current pregnancy and assessed its relationship with the pregnancy that happened hypothetically following the violence while controlling for lifetime IPV and still found a strong relationship. Looking at data in this way enhances chances of predicting the relationship more accurately [49]. Our finding clearly shows the need for maternal health care workers to assess the role of IPV in maternal health.

FFS was associated with a history of miscarriage, any history of negative outcomes in one’s lifetime. It was also associated with a recent unplanned pregnancy and late booking of the recent pregnancy. Although FFS has been researched among young people with associations with STIs being found [16] we do not know of any study that has looked at these relationships in a pregnant or postnatal population. Being initiated into sex through rape or use of force may weaken one’s self-esteem leading to failure to negotiate and plan for sex which ultimately leads to an unplanned pregnancy. Having a low self-esteem may also be associated with booking late which signals a violent and constraining relationship. Further studies are required to look at the pathways from forced first sex, which usually occur in adolescence or earlier, to pregnancy outcomes.

While the study presented significant findings, a few limitations must be highlighted to assist in the interpretation of the results. The cross sectional nature does not allow us to conclude that IPV causes poor maternal health outcomes although we interviewed women retrospectively about their lifetime and past IPV experiences. We can only hypothesise the relationship which prospective studies can confirm such causality. However, since we were able to control for covariates, we believe that the results of this study are indeed meaningful and follow previous trends in highlighting the presence and strength of the relationships reported. The fact that we traced the IPV from its onset around forced first sex, IPV in the lifetime, via the last 12 months before pregnancy up to the recent episodes during the recent pregnancy helps us to conclude that the relationship is indeed illustrative of the interconnectedness of violence and maternal health outcomes.

IPV was self-reported which may lead to underreporting and underestimating IPV because some women may choose to conceal some IPV experiences that they perceive as minor or unimportant to report [9] leading to biasing the results and ultimately resulting in obtaining weaker associations as also seen on associations with most recent pregnancy. However, our methodology has its strengths, for example, increased disclosure of IPV due to privately asking women about violence in clinic settings and using internationally tested tools that describe violence through many acts rather than describing women as abused.

One major strength of this paper is the ability to report on individual types of violence compared to studies that disregarded the types of violence and only reported violence as physical/sexual; or physical, sexual or emotional. Although violence types do not usually occur on their own, unbundling them helps programme implementers to target the specific IPV acts together with established violence drivers. Again, tracing violence and maternal health outcomes from current experiences to lifetime experiences helps to assess the full spectrum of a woman’s life experiences as done in this study. The analysis controlled for covariates and so helped to give a fuller picture of factors that are individually associated with the respective IPV measures.

## Conclusions

The strong relationship between IPV and forced first sex and maternal and newborn health outcomes were described. The paper, through a unique analysis, gives an opportunity to trace a woman’s lifetime experiences of IPV at different stages and linking them to lifetime experiences of maternal health outcomes again at different stages. Our finding that violence in a woman’s life is related to successive maternal health outcomes is a cause for concern that requires concerted prevention efforts by public health providers. Dealing with IPV would reduce negative maternal and newborn health outcomes.

## Abbreviations

aOR: Adjusted Odds Ratio
CI: Confidence Interval
DHS: Demographic and Health Survey
FFS: Forced First sex
HIV: Human immunodeficiency virus
IPV: Intimate partner violence
STI: Sexually transmitted infection
WHO: World Health Organisation
